# The Blockchain Technology Applied in the Development of Real Economy in Jiangsu under Deep Learning

**DOI:** 10.1155/2022/3088043

**Published:** 2022-05-06

**Authors:** Wenquan Shi, Qibao Huang

**Affiliations:** ^1^Faculty of Economics and Management, Suzhou Polytechnic Institute of Agriculture, Suzhou 215008, China; ^2^Academic Committee of Chinese Academy of Management Sciences, Beijing 101199, China

## Abstract

This study focuses on the financing difficulties of small and medium enterprises (SMEs) in China to study the application of blockchain technology in developing the real economy. Deep learning neural network is applied to the vulnerability analysis and detection of smart contracts in blockchain technology by analyzing the connotation of blockchain technology and deep learning. A multiparty joint financial service platform based on blockchain technology is established to help SMEs financing institutions reduce transaction costs, thereby helping them reduce loan interest rates. Finally, Jiangsu Province is studied as a pilot unit. The results show that the Recall and F-score of Bidirectional Neural Network for smart contract vulnerability detection are higher than those of the original neural network. The Recall rate and F-score value of the Wide and Deep model are up to 96.2% and 94.7%, which are higher than those of other vulnerability detection schemes. The Timestamp vulnerability has the highest Recall rate, 94.2%, which can rely on a large amount of valid data to improve detection efficiency. The distribution of financing needs of SMEs in Jiangsu Province from 2020 to 2021 shows that the loan number of SMEs is generally not high. Still, financial institutions and enterprises must spend the same transaction cost. After a technology company in Nanjing made a loan through a blockchain financial service platform, its financing cost decreased by 0.5331%. Blockchain technology has played a great role in the financing process of SMEs, reducing intermediate links and credit costs, and promoting the development of SMEs and the real economy.

## 1. Introduction

The world's major economies are deploying digital technologies, the competition in key core technology fields is becoming increasingly fierce, and the global competitive landscape is also profoundly evolving. With the development of real economy business, both business scale and asset securitization product types have ushered in rapid development. How to better help enterprises, especially small and medium enterprises (SMEs), to obtain lower-cost financing through asset securitization is still a problem that the market needs to explore and solve. With the further support of the policy and the reform of the financial market, asset securitization as a new financing method is increasingly favored by enterprises [1–3]. Due to factors such as their own business-level capability and capital scale, many SMEs in the composition of the real economy has many problems with high operating risks, which makes bank loans to SMEs face excessive credit risks [4–6]. The continuous emergence of new technologies such as the Internet of Things, big data, and cloud computing has brought tremendous changes to traditional industries worldwide, triggering a new round of technological revolution. Blockchain has the characteristics of credit transmission and value transmission, so it is the basic protocol for building the next generation of trusted Internet. It has a very large application space in the economic and social fields [7–9]. Deep learning, as one of the key technologies for the explosion of artificial intelligence, has made breakthroughs in the fields of computer vision and natural language processing, but the specific applications in economics and finance still require more research [10, 11]. Albers and Quedenfeld (2021) [12] pointed out that power consumption is a major cost factor for data centers, which can be reduced by dynamically adjusting the data center's size according to the currently arriving jobs. Suppose the load is low for a long time and power off the server to save energy. Parvin et al. [13] pointed out the need for better control and optimization of energy management combined with renewable energy to improve building energy efficiency and meet the comfort level of indoor environments.

Smart contracts take advantage of the distributed structure of blockchain and are widely used in financial and economic fields by automating peer-to-peer transactions. As a new technology, blockchain can effectively integrate financial resources to analyze and process data, improve the financial system from the direction of data, rules, and applications, improve the efficiency and quality of financial operations and services, and generate new financial formats or services [14–16]. Blockchain technology can help the financial industry identify the credit status of customers, reconstruct the credit system of the financial market, and improve the efficiency of cross-border payments automatically and accurately. Additionally, this technology also poses challenges to the development of the financial industry. Zhang et al. (2020) [17] analyzed blockchain technology and its application in the financial and economic fields, as well as the current situation and challenges, and put forward constructive suggestions to promote the development of blockchain technology in the financial and economic fields. Chang et al. (2020) [18] used blockchain technology for a potential paradigm shift in trade finance. Traditionally, a centralized operating model controlled trade finance and how traders handled business processes. However, the heavy reliance on centralized authority has led to poor performance, lack of flexibility and transparency, and vulnerability to malicious changes. Kowalski et al. (2021) [19] conducted in-depth interviews with industry experts to study how blockchain technology affects the trust relationship between trading partners. The results show that the technology enhances trusting relationships by increasing the security of transactions and data exchange, facilitating expressions of goodwill, improving the efficiency and quality of communication, and increasing the predictability of trading partners. Qiao et al. (2021) [20] introduced cutting-edge blockchain technology from four directions: blockchain systems, consensus algorithms, smart contracts, and scalability. Chen et al. (2021) [21] pointed out that the problem of increasing hidden dangers of network security needs attention, including information leakage and malicious network attacks. Although the research on blockchain technology is complex, the main research direction is still focused on the encryption mode and underlying technology of blockchain. The combination of blockchain technology and deep learning provides new ideas for the application of blockchain technology in economic development.

Based on these questions, literature studies and surveys are employed. By analyzing deep learning and blockchain technology, it is applied in the financial field. The innovation lies in the use of deep learning neural networks in smart contract vulnerability detection, which improves the Accuracy and Recall rate of vulnerability detection. On this basis, a financial service platform based on blockchain technology is established. The platform is used to solve the financing difficulties of SMEs and provides new ideas for the development of the real economy in Jiangsu Province.

## 2. Related Work

Blockchain is a technological revolution that commodifies trust. Smart contracts are the tools that help people realize this revolution. It uses smart contracts to develop and implement a new paradigm of cryptoeconomics. Cryptoeconomics is one of the most interesting ways to achieve trust. Combining cryptographic determinism with the design of economic mechanisms to incentivize specific behaviors allows for the realization of novel systems. Systems are not possible until blockchain technology is developed. Blockchain is a peer-to-peer (P2P) network built on the Internet. Under the current business model, continuous recording of transactions is a core function of every company. These records track past actions and performance and guide future planning.

Blockchain technology is a revolutionary new protocol for sharing and updating information by linking ledgers or databases in a decentralized, P2P open-access network. Blockchain is designed to ensure that data is stored and updated in a secure, tamper-proof, and irreversible manner. Although the technology is in its infancy, blockchain research is developing rapidly in different fields. The circular economy also focuses on enhancing sustainability and social responsibility alongside economic growth. Upadhyay et al. (2021) [22] reviewed the current and potential contributions of blockchain technology to the circular economy through the lens of sustainability and social responsibility. Through identification, different studies on the blockchain are collated and organized to contribute to the Industry 4.0 literature. Its positive and potential impact on the ethics plan is highlighted. The circular economy concept has great potential to overcome the shortcomings of current economic activities and promote sustainable development. But the concept also faces challenges in realizing its potential. Blockchain technology has been suggested as a possible key solution to overcome the current barriers to implementing circular economy concepts. Böckel et al. (2021) [23] explored the emerging research area of blockchain in the circular economy and examined current developments from research and practice. Patterns of interest and opportunities for research and practice are identified using systematic literature reviews to develop and practice gaps. Mutual blind spots that any field needs to address are revealed. Yildizbasi (2021) [24] discussed eliminating the problems encountered in the energy grid management process, the blockchain concept, and its integration with renewable energy systems. The application of blockchain technology in the development of the real economy can provide reliable solutions to current economic development problems.

Recently, blockchain has emerged as a research trend with potential applications in a wide range of industries and contexts. One particularly successful blockchain technology is smart contracts. It is widely used in commercial settings, for example, high-value financial transactions. However, this has security implications. This technology has the potential to gain financial benefits from security incidents, such as identifying and exploiting vulnerabilities in smart contracts or their implementations. Ethereum is the most active and high-profile platform [25] and is currently the second-largest blockchain platform by market capitalization and the largest smart contract blockchain platform. Smart contracts can simplify and speed up the development of various applications, but they also bring some problems. For example, smart contracts are used to commit fraud. Buggy contracts are deliberately developed to undermine fairness. Many duplicate contracts waste performance for no real purpose. Hu et al. (2021) [26] proposed a transaction-based Ethereum smart contract classification and detection method to solve these problems. Over 10,000 smart contracts are collected in Ethereum and focus on smart contracts and user-generated data behavior. The manual analysis identified four behavioral patterns from the transactions. These patterns can be used to distinguish the differences between different types of contracts. A key issue in smart contract security is efficient and fast vulnerability detection in smart contracts. Most of the existing detection methods can only detect vulnerabilities existing in contracts, and it is difficult to identify their types. Also, they are less scalable. Huang et al. (2022) [27] developed a multitask learning-based smart contract vulnerability detection model to address these issues. The detection ability of the model is improved, and the detection and identification of vulnerabilities are realized by setting auxiliary tasks to learn more directional vulnerability features. The research on vulnerability detection of smart contracts has also achieved remarkable results. However, there are few studies on its combination with deep learning, and the results are lacking. Deep learning technology is used in smart contract vulnerability detection, which greatly improves detection efficiency and fills the research gap in this area.

## 3. Materials and Methods

### 3.1. Blockchain Technology under Deep Learning

**Figure 1:**
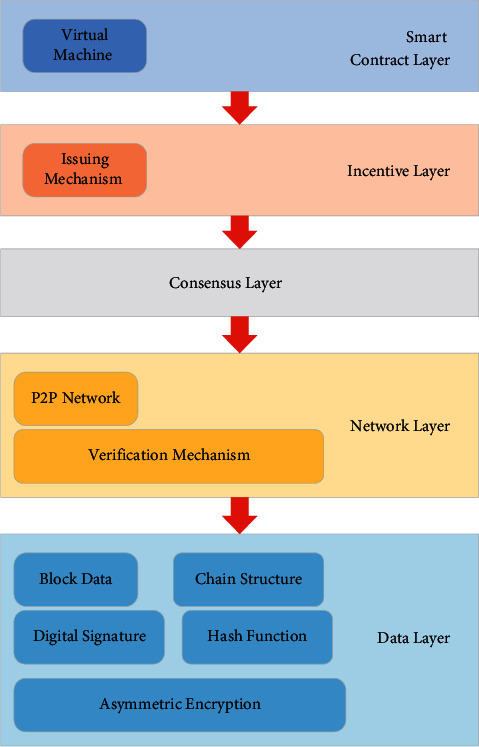
Common architecture of blockchain.

Blockchain technology applies a series of distributed computing, data structures, and encryption mechanisms to create a secure online transaction system. In this trading system, buyers and sellers can directly conduct secure transactions. The content of the transaction is completely recorded and cannot be tampered with. The system eliminates all intermediate links. All intermediary chains involved in digitized transactions will be affected. For example, a large part of banks and many financial businesses serve as an intermediary to provide services to buyers and sellers. If buyers and sellers can connect directly through a secure platform, then the existence of these services is unnecessary [28]. As a new type of digital database for data storage, blockchain can not only ensure that transaction information in the supply chain is not tampered with and reduce financing costs but also improve financing efficiency. As a distributed database that maintains the same ledger in multiple locations that are not trusted by a specific algorithm, the blockchain consists of a chain structure with Timestamps. Each block on the chain carries the entire transaction record of the entire network. Its core technologies include consensus algorithms, distributed storage, asymmetric encryption, and P2P network technology. The common architecture of blockchain is shown in Figure 1.

**Figure 2:**
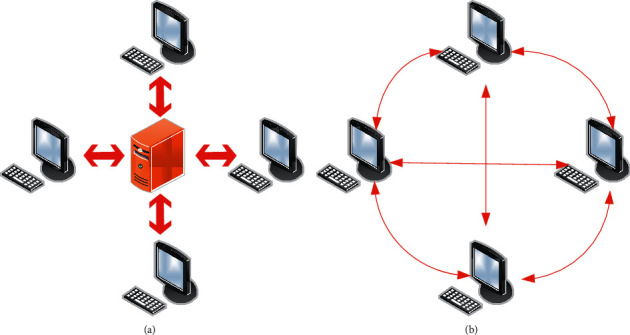
Comparison of central and distributed storage architectures: (a) central; (b) distributed.

In Figure 1, the consensus algorithm, as the consensus protocol at the bottom of the blockchain, can ensure the synchronization, transparency, and sharing of information between nodes. Data storage in blockchain is not managed by hosts but by nodes in a decentralized blockchain network. Each node has the same administrative authority, which can ensure data integrity. Only when more than half of the nodes on the blockchain are destroyed can data be tampered with. So, it is very safe. The comparison of central and distributed storage architecture is shown in Figure 2.

**Figure 3:**
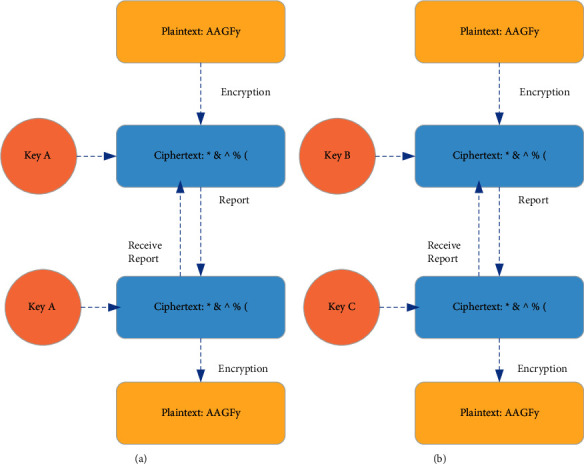
Comparison of symmetric and asymmetric encryption methods: (a) symmetric; (b) asymmetric.

In Figure 2, asymmetric encryption algorithms are commonly used in blockchain. Different from symmetric encryption, its core is to use a pair of keys to ensure the security of the data transmission process, that is, the public key and the private key. Both public and private keys can encrypt and decrypt information. After one party encrypts, the other party can decrypt it. Because the encryption and decryption keys are not the same, it is asymmetric encryption. The comparison between symmetric and asymmetric encryption processes is shown in Figure 3.

In Figure 3, since each node in the blockchain network is equal, the information access and resource sharing of each node does not need to be unified by the centralized host. Any two nodes can have point-to-point communication. Deep learning is a method in machine learning based on representational learning of data. Observations can be represented in various ways, as a vector of intensity values for each pixel or more abstractly as a series of edges, regions of a specific shape, and so forth. Instead, it is easier to learn tasks from examples using some specific representation. Although deep learning can demonstrate very powerful AI capabilities through learning and training, deep learning cannot yet understand the so-called “logical relationship.” For example, although deep learning can identify women, the elderly, and children, it cannot identify concepts such as mothers, daughters, and grandma. It is even more impossible to understand the relationship between these different women. The reason is that the learning training samples of deep learning do not include related concepts. The blockchain is a management technology for processing data, and all data recorded on the chain have a logical sequence. If the data on the blockchain is purposely set as training samples with logical order, then deep learning can learn from the data on the blockchain. The snapshot information of the data is combined with the process information of the data, and it can learn more abstract concepts. On the surface, blockchain and deep learning are two relatively distant technical fields. The unique characteristics of blockchain itself can still provide deep learning with support for learning and training data. Of course, the smart contract mechanism on the blockchain can also relate to deep learning so that the output of deep learning can trigger the smart contract, or the execution result of the smart contract can be used as the input of deep learning. Therefore, blockchain and deep learning have a space for docking and cooperating.

### 3.2. Smart Contract Vulnerability Detection Based on Deep Learning

**Figure 4:**
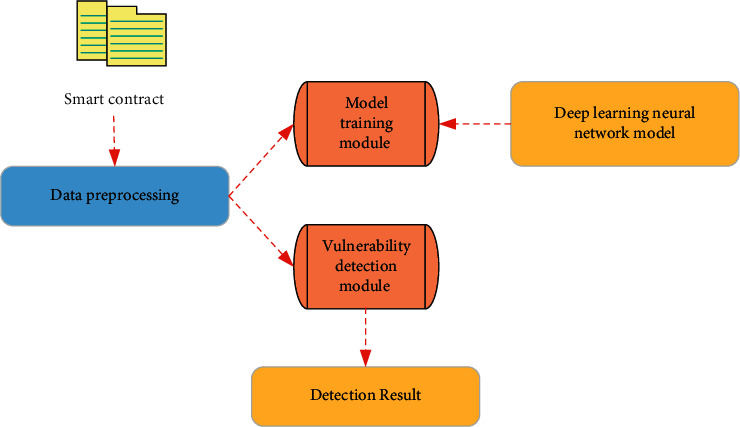
Smart contract vulnerability detection frameworks based on deep learning.

As a piece of code that runs automatically on the blockchain, a smart contract can complete tasks according to preset rules. Manual programming will inevitably lead to loopholes, especially in the financial field, and the losses caused by loopholes are unpredictable. Such vulnerabilities are classified into code layer vulnerabilities, virtual machine layer vulnerabilities, blockchain layer vulnerabilities, and denial of service vulnerabilities due to different causes. Solidity language, as the main programming language for smart contracts, may cause vulnerabilities due to improper use of the language, such as arithmetic overflow and underflow. Mutation errors in smart contracts can cause programs not to perform as expected. Different blockchain systems will cause differences in application fields and characteristics, and the blockchain network cannot be used normally due to denial-of-service attacks by attackers. Vulnerabilities caused by these reasons need to be modeled for detection. Aiming at the problems of high false-positive rate, long time, and low degree of automation in automatic auditing technology in smart contracts, a smart contract vulnerability detection scheme based on deep learning is established, as shown in Figure 4.

In Figure 4, the system consists of three stages: preprocessing, model training, and vulnerability detection modules. The preprocessing module processes the code in the smart contract, slices the source code according to function calls and function point features, and labels the slices so that a series of functionally complete program slices with vulnerability labels are obtained. The program slices are then converted into vectors that deep learning neural networks can use. The model training part of vulnerability detection is used as a key step, which is to configure a specific deep learning algorithm for the smart contract vulnerability detection framework and train a specific classification model. During the training process, the model and algorithm parameters are adjusted according to the requirements. The deep learning model with the optimal parameter combination is obtained. The training module neural network selects Recurrent Neural Network (RNN), Long Short-Term Memory (LSTM), or Wide and Deep learning model. This study chooses a bidirectional LSTM neural network as the neural network in the Deep component.

Assume that the number of samples of known smart contracts is *N*, and the number of types of contract vulnerabilities is denoted as *T*. Each sample is marked by vulnerability type, set to *t*, 1 ≤ *t* ≤ *T*. The vector of samples marked with *t*-vulnerability is denoted as *X*_*t*_. Then, in the Forward layer, starting from the first vulnerability, the hidden layer output of each vulnerability calculated forward is denoted as *h*. Then, the output obtained by the *t*-th vulnerability is expressed as *h*_*t*_:

(1) $$\begin{array}{c} h_{t}=\sigma \left(W_{1}X_{t}+W_{2}h_{t-1}+b\right), \end{array}$$

where *σ* represents the sigmoid activation function, shown in the following equation:

(2) $$\begin{array}{c} f\left(x\right)=\frac{1}{1+e^{-x}}, \end{array}$$

and *W*_*i*_(*i*=1,2,3,4,5,6) represents the weight preset by the system. *b* represents the bias value preset by the system. In the Backward layer, the output result of each vulnerability backward hidden layer obtained from the backward calculation from the *t*-th to the first vulnerability is denoted as *h*. Then, the result obtained by the backward calculation of the *t*-th vulnerability is expressed as *h*_*t*_:

(3) $$\begin{array}{c} h_{t}=\sigma \left(W_{3}X_{t}+W_{4}h_{t+1}+b\right). \end{array}$$

The output is the word vector and the discriminant *z*_*t*_ by the vulnerability type through the training of the Deep component. Its decision set is denoted as *Z*={*z*_1_, *z*_2_, *z*_3_,…, *z*_*t*_,…*z*_*T*_}. The determination process is shown as follows:

(4) $$\begin{array}{c} z_{t}=g\left(W_{5}X_{t}+W_{6}h_{t}+b\right), \end{array}$$

where *g* represents the ReLU activation function, shown in the following equation:

(5) $$\begin{array}{c} f\left(x\right)=\max\left(0,1\right). \end{array}$$

The Wide component is to input the training set into the model for training, as shown in the following equation:

(6) $$\begin{array}{c} y_{t}=g\left({\text{WX}}_{\text{wide}}+B\right). \end{array}$$

The obtained set of predictions is denoted as *Y*={*y*_1_, *y*_2_, *y*_3_,…, *y*_*T*_}. Among them, *X*_wide_ represents the training set vector and *B* represents the preset bias. Then, the training results of the Deep component and the Wide component are weighted to obtain the final prediction result *p*_*t*_, as shown in the following equation:

(7) $$\begin{array}{c} p_{t}=\sigma \left(W_{\text{deep}}^{T}\left[z_{t}\right]+W_{\text{wide}}^{T}\left[y_{t}\right]+B\right), \end{array}$$

where *W*_deep_^*T*^ and *W*_wide_^*T*^ represent the weights of Deep components and Wide components, respectively. Then, the deviation of the current model is calculated by the deviation of the predicted value *p*_*t*_ and the vulnerability label, as shown in the following equation:

(8) $$\begin{array}{c} \text{Loss}=\sum_{1}^{G}\sum_{1}^{T}\left(p_{t}-t\right). \end{array}$$

In equation (8), *t* represents the vulnerability annotation. *t* = 1 when there exists the vulnerability, and *t* = 0 when there is no such vulnerability. ∑_1_^*T*^(*p*_*t*_ − *t*) represents the error of a single smart contract. Finally, iterative training is performed by gradient descent until the preset weight is corrected to be smaller than the preset value. Gradient descent is shown in the two following equations:

(9) $$\begin{array}{c} W=\alpha \times \text{Loss}\times q, \end{array}$$

(10) $$\begin{array}{c} \alpha =\left\{\begin{array}{c} 0.1\\ 0.01\\ 0.001 \end{array}\right), \end{array}$$

where Loss × *q* stands for gradient. Finally, *α* vulnerability detection module is used for detection.

Common evaluation indicators in the field of machine learning are Accuracy, Recall, Precision, F-score, True Negative Rate (TNR), False Positive Rate (FPR), False Negative Rate (FNR), and True Positive Rate (TPR). The calculation method is as follows:

(11) $$\begin{array}{c} \text{Accuracy}=\frac{\text{TP}+\text{TN}}{\text{TP}+\text{FP}+\text{FN}+\text{TN}}\text{.} \end{array}$$

TP means True Positive, where the predicted result is positive, and the actual result is positive. FN stands for False Negative, where the predicted result is negative, but the actual result is positive. FP means False Positive, where the predicted result is positive, but the actual result is negative. TN stands for True Negative, where the predicted result is negative, and the actual result is negative.

(12) $$\begin{array}{c} \text{Recall}=\frac{\text{TP}}{\text{TP}+\text{FN}},\\ \text{Precision}=\frac{\text{TP}}{\text{TP}+\text{FP}},\\ F-\text{score}=2\times \frac{\text{Precision}\times \text{Recall}}{\text{Precision}+\text{Recall}},\\ \text{TNR}=\frac{\text{TN}}{\text{FP}+\text{TN}},\\ \text{FPR}=\frac{\text{FP}}{\text{FP}+\text{TN}},\\ \text{FNR}=\frac{\text{FN}}{\text{TP}+\text{FN}}\text{,}\\ \text{TPR}=\frac{\text{TP}}{\text{TP}+\text{FN}}\text{.} \end{array}$$

Google's TensorFlow 2.0 deep learning framework is chosen for experimentation. The operating environment is set to Intel(R) Core(TM) i7-8550U 1.80 GHz processor, 32G RAM memory, 8G video memory, and kernel Linux version 4.8.0–52-generic. The dataset comes from web platforms and smart contract vulnerabilities that have been discovered. There are 40135 smart contract samples in total. The samples are divided into the training set and test set with the ratio of 8 : 2. The composition of the sample distribution is shown in Table 1.

### 3.3. Application of Blockchain Technology in Cross-Border Financing of SMEs in Jiangsu Province

The cross-border financing of SMEs has greater financing risks due to the problem of information asymmetry. Due to the small scale of SMEs, there is a problem of nonstandard financial management. It is difficult for enterprises to collect information, and it is difficult to calculate credit lines in banks. Traditional trade finance cannot meet the requirements of the digital economy era in terms of trust mechanism construction and risk control. The advantages of blockchain technology information, such as nontampering, distributed ledger management, and information sharing, can provide technical support for financial trade. Taking Jiangsu Province as an example, SMEs account for most of the real economy. Not only can a credible trading environment solve the cross-border financing problem of SMEs but also it improves the status quo of the overall real economy. Some problems need to be solved urgently in Jiangsu Province, such as the time-consuming process of applying for loans for SMEs and the cumbersome examination and approval procedures.

**Figure 5:**
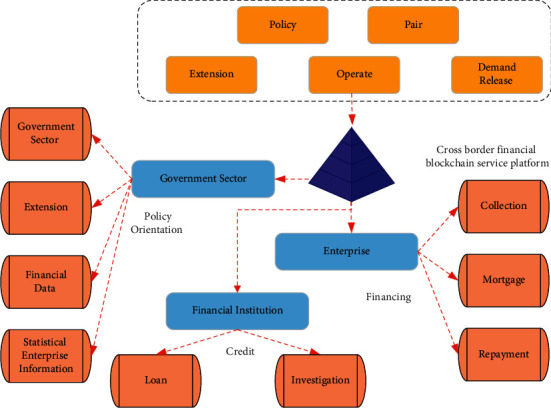
The roles and functions of the cross-border financial blockchain service platform.

Cross-border financial blockchain services are the product of blockchain technology and cross-border financial services. Blockchain can create a trusted information exchange platform between businesses and financial institutions. Blockchain can improve the audit efficiency of financial institutions and increase the trust in SMEs through data sharing and optimizing the financing process of financial institutions. Thus, financial institutions are willing to provide loan businesses for SMEs. The adopted cross-border financial blockchain service platforms include financial institutions, SMEs, and foreign exchange administrations. The composition and functions of platform roles are shown in Figure 5.

In Figure 5, all parties share data integration resources through the platform, which is conducive to alleviating the dilemma of cross-border financing and assisting the long-term development of SMEs. As the provider of funds in financing transactions, financial institutions are responsible for uploading the financing information of SMEs to the blockchain financial platform for sharing. This will help improve the efficiency of loan approvals of other financial institutions and can also effectively prevent repeated financing. Additionally, the information obtained by financial institutions in conducting due diligence on SMEs should also be shared on the platform promptly. Such an approach is conducive to reducing the transaction costs of financial institutions and the financing costs of SMEs. SMEs need to agree that the service platform can use the data information of the enterprise, including the foreign exchange payment information, industrial and commercial information, tax information, and transaction logistics information of the foreign exchange administration to realize the platform data sharing. Additionally, enterprises are obliged to cooperate with financial institutions' due diligence to increase credit information for enterprises and facilitate the loan review of financial institutions. Additionally, the State Administration of Foreign Exchange needs to cooperate in providing export declaration data. The tax department provides the tax filing data of the enterprise. The National Port Logistics Office provides logistics information of enterprises and helps financial institutions conduct multidimensional data analysis on SMEs to reduce business risks. The platform brings the three parties together for integration. In SME lending, it is no longer necessary to seek financing opportunities from financial institutions. Financial institutions do not need to verify the authenticity of SMEs credit information repeatedly, and government departments also have reliable supervision tools. Such a comprehensive service platform can effectively improve the financing efficiency of SMEs and reduce the lending risk of financial institutions.

**Figure 6:**
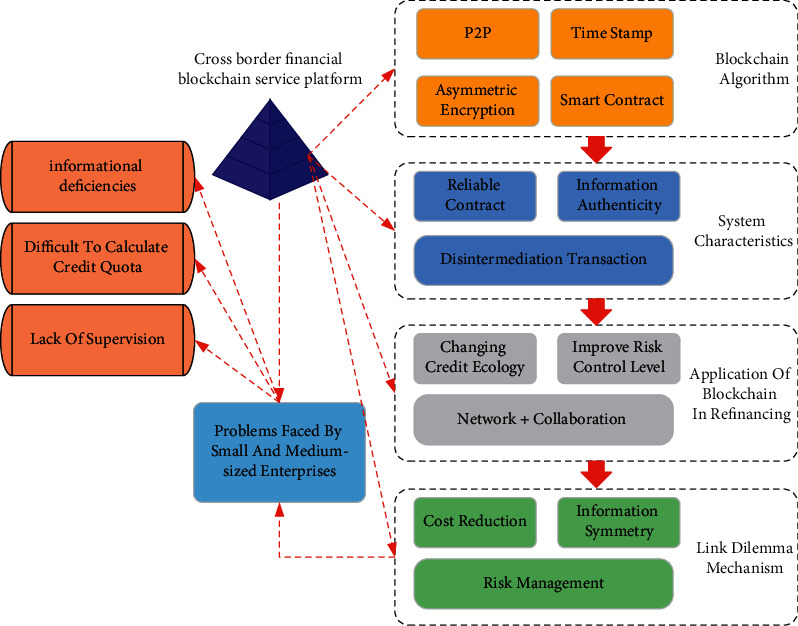
Models of cross-border financing applications for SMEs.

In the cross-border financial blockchain service platform, the credit information of SMEs' cross-border financing has undergone informatization processing and credit approval. This can better solve the problem of information asymmetry between banks and enterprises, thereby improving the availability of cross-border financing for SMEs. Blockchain technology solves cross-border financing problems for SMEs through cross-border financial blockchain service platforms. System credit can be formed based on the smart contract algorithm in blockchain technology. This kind of credit helps blockchain technology to establish advantages in the application of SMEs in cross-border financing. The problems faced by SMEs in cross-border financing are finally solved through the mitigation mechanism of blockchain technology to the cross-border financing dilemma of SMEs. The application model is shown in Figure 6.

In Figure 6, before the cross-border financial blockchain platform is used, banks need to use the enterprise electronic port card or check the authenticity of the enterprise customs declaration through the customs website for trade financing. It takes a long time to verify the customs form, and it is impossible to know whether other banks have used it. The cost of information delay time caused by this is the cost of the loan process. The blockchain service platform eliminates these costs and can offer lower loan interest rates.

Although blockchain technology can help SMEs reduce costs, it cannot provide SMEs with the credit they deserve. This technology can only be used as a technical means to ensure the stability of credit circulation. The credit in the cross-border financing business of SMEs ultimately depends on the ability of the enterprise itself to determine whether the loan can be successful. Therefore, if SMEs have irregular management systems and imperfect financial systems, even with the help of blockchain technology, the credit rating of SMEs is still insufficient, and they still cannot obtain mortgage loans from financial institutions.

Jiangsu Province is used as a pilot unit. From 2020 to 2021, the loan needs of some SMEs in Jiangsu Province are investigated. The use of blockchain financial services platforms for lending is encouraged. A science and technology company in Nanjing, Jiangsu, is taken as an example, and a specific usage analysis is carried out. The loan cost situation is compared.

## 4. Results and Discussion

### 4.1. Comparison of Vulnerability Detection Results of Different Neural Networks

**Figure 7:**
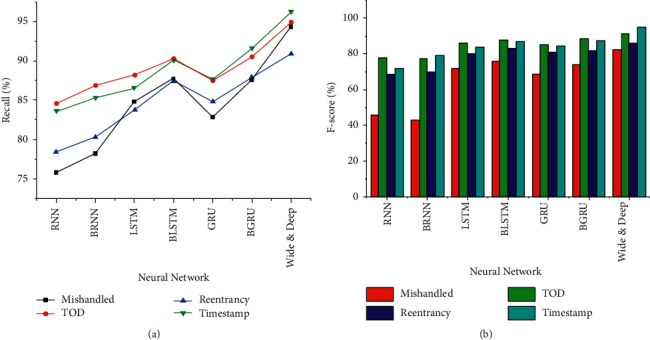
Vulnerability detection's Recall and F-score comparison of different neural network training. (a) Recall; (b) F-score.

Compare several neural networks commonly used in smart contract vulnerability detection: RNN, Bidirectional Recurrent Neural Network (BRNN), LSTM, Bidirectional Long Short-Term Memory (BLSTM), Gated Recurrent Unit (GRU), and Bidirectional Gated Recurrent Unit (BGRU); the Recall and F-score results of vulnerability detection are shown in Figure 7.

In Figure 7, the Recall and F-score values of Bidirectional Neural Network vulnerability detection are higher than those of the original neural network, indicating that the Bidirectional Neural Network is more effective when smart contracts are used for vulnerability detection. This is because, during the training process of the one-way neural network, the next output is only affected by the previous output. The Bidirectional Neural Network is jointly determined by the output results several times before and after. When a smart contract detects a vulnerability, it correlates the historical output and future output, and the context is more closely related. The Recall rate and F-score value of the Wide and Deep model are up to 96.2% and 94.7%, which are higher than other neural network models. This is because the Wide and Deep model is trained by combining two components, which has not only the memory ability of the Wide component but also the generalization ability of the Deep component. During training, the parameters of both models are optimized simultaneously. This improves the model's accuracy for classification and compatibility with new vulnerabilities.

**Figure 8:**
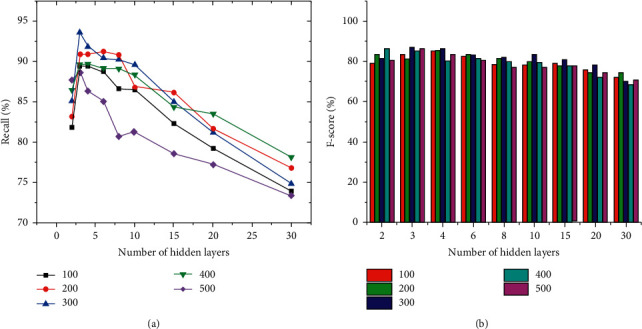
Comparison of Recall and F-score for vulnerability detection with different number of hidden layers and number of nodes. (a) Recall; (b) F-score.

The number of hidden layers and the number of nodes in each layer will affect the detection results. Set the number of nodes in the experiment to 100, 200, 300, 400, and 500. The number of hidden layers is selected as 2, 3, 4, 6, 8, 10, 15, 20, and 30, and the changes of the results are observed. The comparison between Recall and F-score is shown in Figure 8.

In Figure 8, when the number of hidden layers is 3 and the number of nodes is 300, the maximum Recall value is 93.7%. Then, as the number of hidden layers increases, the Recall value gradually decreases. This is because the neural network model is composed of multiple RNN units superimposed on each other. The more layers there are, the disappearance of the gradient will occur, which affects the Recall value of the test result. Similar conclusions are obtained for the F-score value. Therefore, the number of hidden layers is set to 3.

**Figure 9:**
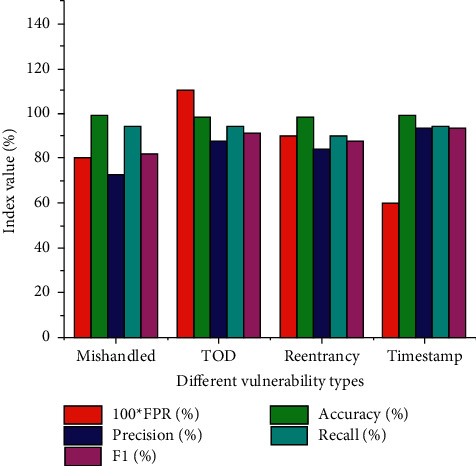
Comparison of detection results of different vulnerability types.

In the vulnerability detection framework of the Wide and Deep model, the comparison of detection results of different vulnerability types is shown in Figure 9.

In Figure 9, the Recall rate for all vulnerability types is higher than the precision rate. Smart contracts cannot be tampered with, and their parameters are adjusted—the higher the Recall rate, the lower the Precision rate. Among them, the Accuracy rate of mishandled vulnerability detection is the lowest, only 72.4%, and the F1 value is 81.8%, but the Accuracy rate reaches 99.1%, which can effectively detect vulnerabilities.

The Recall rate of all vulnerabilities is around 90%, the highest in the TOD, with a Recall of 94.7%.

**Figure 10:**
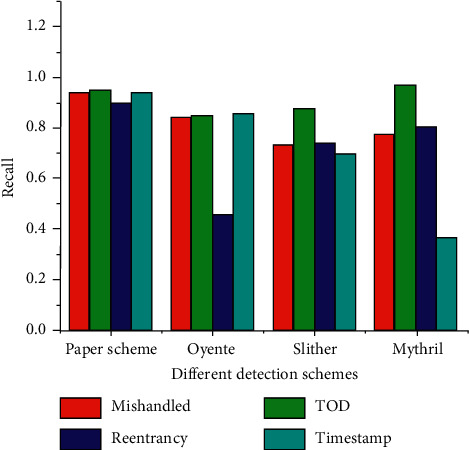
Comparison of different vulnerability detection schemes.

This method is compared with the effects of Oyente, Slither, and Mythril, as shown in Figure 10.

In Figure 10, the detection Recall rate of this scheme in various vulnerability types is higher than those in other schemes. The highest Recall rate of Timestamp vulnerability is 94.2%, which can rely on a large amount of valid data to improve detection efficiency.

### 4.2. The Effect of SMEs in Jiangsu Province Using Blockchain Financial Service Platforms

**Figure 11:**
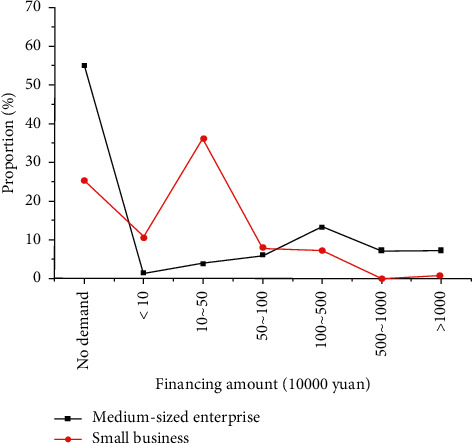
Distribution of financing demands of SMEs in Jiangsu Province in 2020-2021.

The distribution of financing needs of SMEs in Jiangsu Province from 2020 to 2021 is shown in Figure 11.

In Figure 11, the enterprises with financing amounts between 1 million and 5 million yuan are the most, accounting for 13.07%. Small business financing is distributed to nearly 50% of the companies with less than 500,000.

Compared with large enterprises, the loan amount of SMEs in Jiangsu Province is generally not high, but financial institutions and enterprises must spend the same transaction cost. Using blockchain financial service platforms in the credit business can effectively reduce these costs. The transaction cost of financial institutions is reduced, and the financing cost of SMEs that finance on the cross-border financial blockchain service platform will naturally follow.

**Figure 12:**
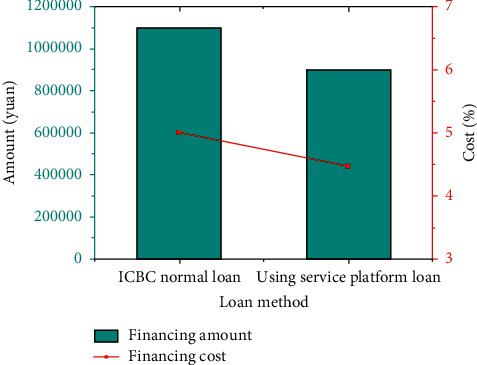
Comparison of loan financing costs for a technology company.

A comparison of the cost of loan financing by a technology company in Jiangsu Province using this service platform is shown in Figure 12.

In Figure 12, the financing cost of the tech company decreased by 0.5331% after adopting the blockchain financial service platform for loans.

The reason why the financing obtains lower loan interest rates is that the cost of lending to SMEs by financial institutions in the service platform is reduced. Then, the loan interest rate of SMEs will naturally drop. Therefore, the advantage of the cross-border financial blockchain service platform to alleviate the cross-border financing dilemma of SMEs is to combine all the data information of government departments, financial institutions, and SMEs. The transparency and sharing of information enable the platform to use automated processing procedures to achieve economies of scale and reduce transaction costs. Ultimately, financing costs are reduced for SMEs. The development of SMEs drives the further development of the real economy.

## 5. Conclusion

SMEs are an important part of China's real economy. From the perspective of development history, they have made great contributions to tax payment, labor employment, technological innovation, and even social stability. However, the difficulty and high cost of financing have always restricted the healthy and sustainable development of SMEs. With the advancement of science and technology, the development of deep learning and blockchain technology provides new directions for financing. Through deep learning in smart contract algorithms in blockchain technology, neural networks can help smart contract algorithms perform vulnerability analysis. A financial service platform based on blockchain technology is established, which can help SMEs financing institutions save costs and reduce loan interest rates, and has been verified in some SMEs in Jiangsu Province. As a result, the Recall and F-score of Bidirectional Neural Network for smart contract vulnerability detection are higher than those of the original neural network. The Recall rate and F-score value of the Wide and Deep model are up to 96.2% and 94.7%, which are higher than those of other vulnerability detection schemes. Timestamp vulnerability Recall rate reaches 94.2%, which can improve detection efficiency by relying on a large amount of valid data. As an important part of the real economy of Jiangsu Province, SMEs in Jiangsu Province will also drive the development of the real economy in Jiangsu Province. The application of blockchain technology in the financial field is very effective. However, there are still some deficiencies to be improved. For example, only four common smart contract vulnerabilities are analyzed in the smart contract security vulnerability detection process, and more instances should be used for verification. The established financial service platform has only been piloted in Jiangsu Province. In future research, SMEs across China will verify the application of blockchain technology in the financial industry, which will bring positive changes to the real economy.

## Figures and Tables

**Table 1:** Sample distribution of smart contract vulnerabilities.

Vulnerability type	Vulnerability sample	No vulnerability samples	Exception handling vulnerability	Transaction order dos (TOD)	Reentrancy vulnerability	Timestamp
Sample set	6464	25644	708	2,456	1644	2536
Test set	1616	6411	177	614	411	634

## Data Availability

The research data used to support the findings of this study are included within the article.
